# Risk Reduction Assessment of *Vibrio parahaemolyticus* on Shrimp by a Chinese Eating Habit

**DOI:** 10.3390/ijerph20010317

**Published:** 2022-12-25

**Authors:** Huan Xu, Jing Liu, Mengqi Yuan, Cuifang Tian, Ting Lin, Jiawen Liu, Olivera Castro Osaris Caridad, Yingjie Pan, Yong Zhao, Zhaohuan Zhang

**Affiliations:** 1 College of Food Science and Technology, Shanghai Ocean University, Shanghai 201306, China; m200300891@st.shou.edu.cn (H.X.); d210300079@st.shou.edu.cn (J.L.); m200300881@st.shou.edu.cn (M.Y.); m200300895@st.shou.edu.cn (C.T.); m200311008@st.shou.edu.cn (J.L.); osarisolivera@yahoo.com (O.C.O.C.); yjpan@shou.edu.cn (Y.P.); 2 Greentown Agricultural Testing Technology Co., Ltd., Hangzhou 310051, China; wx4linting@126.com; 3 Laboratory of Quality and Safety Risk Assessment for Aquatic Products on Storage and Preservation (Shanghai), Ministry of Agriculture and Rural Affairs, Shanghai 201306, China; 4 Shanghai Engineering Research Center of Aquatic-Product Processing and Preservation, Shanghai 201306, China

**Keywords:** Chinese eating habit, *Vibrio parahaemolyticus*, risk reduction assessment, ready-to-eat shrimp

## Abstract

In China, a traditional perspective recommended that consuming seafood should be mixed or matched with vinegar, because people thought this traditional Chinese eating habit could reduce the risk of pathogenic microorganism infection, such as *Vibrio parahaemolyticus* induced diarrhea. However, this empirical viewpoint has not yet been evaluated scientifically. This study conducted a simplified quantitative microbiological risk assessment (QMRA) model, which was employed to estimate the risk reduction of *V. parahaemolyticus* on ready-to-eat (RTE) shrimp by consuming with vinegars (white vinegar, aromatic vinegar, or mature vinegar). Results showed the reduction of *V. parahaemolyticus* density on RTE shrimp after consuming with white vinegar, aromatic vinegar and mature vinegar was respectively 0.9953 log CFU/g (90% confidence interval 0.23 to 1.76), 0.7018 log CFU/g (90% confidence interval 0.3430 to 1.060) and 0.6538 log CFU/g (90% confidence interval 0.346 to 0.9620). The infection risk of *V. parahaemolyticus* per meal in this QMRA model was quantified by a mean of 0.1250 with the standard deviation of 0.2437. After consuming with white vinegar, aromatic vinegar, and mature vinegar, the mean infection risk of *V. parahaemolyticus* on shrimp decreased to 0.0478, 0.0652, and 0.0686. The QMRA scenarios indicated significant reductions in infection risk when eating RTE shrimp by the Chinese eating habit (consuming with vinegar). This good eating habit should be recommended to promote the spread of around the world.

## 1. Introduction

*Vibrio parahaemolyticus*, the leading foodborne pathogen in China, is frequently isolated from a variety of seafood [1,2]. its salinity and fear of acid, in 3–5% salt water can multiply rapidly, but in the pH below 6 acidic conditions is poor growth [3,4]. Acute gastroenteritis brought on by a *V. parahaemolyticus* infection might present as diarrhea, headache, vomiting, nausea, and abdominal cramps [5,6]. One of the most significant types of seafood, shrimp is also in high demand on the global market. However, it is frequently linked to outbreaks of *V. parahaemolyticus*, particularly during the warm seasons [7], which may be brought on by a high incidence of the virus in shrimps. That presented possible threats to the public’s health and seafood safety.

The most preferred method for reducing the risk of infection from *V. parahaemolyticus* on shrimp was thermal processing. Additionally, customers are becoming more and more enamored with cooked, ready-to-eat shrimp due to their convenience and flavor. However, due to cross-contamination from subpar production methods and careless eating and handling habits, ready-to-eat shrimp was also easily infected with *V. parahaemolyticus* [8]. In China, a traditional perspective recommended consuming fish combined with vinegar, since it was believed that this traditional Chinese eating habit might lower the risk of infections from harmful microorganisms found in seafood. However, up to now, this empirical viewpoint has not yet been proved scientifically. Quantitative microbiological risk assessment (QMRA) was considered as a scientific and internationally accepted tool to evaluate the infection risks of foodborne pathogens [9,10,11]. The QMRA model was widely employed to estimate the infection probability of pathogenic bacteria in different foods, such as *Listeria monocytogenes* in ready-to-eat food [12], *Salmonella* spp. in eggs [13], and *V. parahaemolyticus* in seafood [14,15,16,17,18] etc. In addition, it is also a useful tool to quantify the reduction in potential risks of pathogens by sterilization techniques. However, to the best of our knowledge, this approach has not been used to assess the effect of an eating habit on the microbiological infection risk.

Therefore, this study firstly conducted a simplified QMRA model to simulate the circumstance of consuming seafood with vinegar, and attempted to calculate the reduction in infection risk of *V. parahaemolyticus* in shrimp by this kind of Chinese eating habit. The three most comment vinegars in China, white vinegar, aromatic vinegar, and mature vinegar have been chosen in this study. A Monte Carlo simulation was applied to describe the uncertainty and variability of the influence of consuming with vinegar on reduction of *V. parahaemolyticus* concentration. This study aims to promote the adoption of this healthy eating practice worldwide by providing scientific proof that this Chinese eating practice does lower the risk of *V. parahaemolyticus* in shrimp.

## 2. Materials and Methods

### 2.1. Bacterial Strain and Culture Preparation

The pathogenic *V. parahaemolyticus* O3:K6 strain is a pandemic strain currently preserved at our laboratory at −80 °C in Tryptone soy broth (TSB, Beijing Land Bridge Technology Company Ltd., Beijing, PR China) containing 3% NaCl (pH 8.0) with addition of 25% (*v/v*) sterilized glycerol as a cryoprotector.

To prepare the inoculum culture, the stock *V. parahaemolyticus* culture was transferred to TSB plus 3% NaCl and incubated at 37 °C for 18–20 h. After two passages, the suspension from the last culture was centrifuged at 4000 rpm for 10 min at room temperature, the supernatant was discarded and the absorbance was adjusted with fresh sterile Phosphate Buffer Solution (PBS) and the absorbance was determined by colony counting until the bacterial load was approximately 10^9^ CFU/mL.

### 2.2. Preparation of Artificial Contaminated Shrimp Samples

Shrimp samples (10 ± 1 g per) were purchased from a local supermarket in Shanghai. The shrimp samples were stored at −20 °C, and thawed at 4 °C overnight before treatment.

The shrimp samples were randomly selected and dipped into a *V. parahaemolyticus* O3:K6 suspension containing ~10^9^ CFU/mL with shaking for 20 min. The whole shrimp samples were then air-dried in a biosafety hood for 20 min to allow for bacterial attachment. This treatment insured a uniform distribution of *V. parahaemolyticus* on the shrimp (~10^6^ CFU/g).

### 2.3. Treatment of Shrimp Samples with Chinese Traditional Vinegar

Three Chinese traditional vinegars, white vinegar, aromatic vinegar, and mature vinegar, were purchased from a local supermarket in Shanghai. The major components were listed in Table 1. Each group of five inoculated shrimps was immersed in 500 mL of each condiment for 5, 10, 15, 30, 60, 90 and 120 s, respectively. Inoculated samples treated with sterile 0.85% saline solution were used as control.

After vinegar treatment, shrimp samples were placed in a sterile 400 mL filter stomacher bag (Beijing Land Bridge Technology Company Ltd., Beijing, PR China) with 100 mL of sterile alkaline peptone water (APW, Beijing Land Bridge Technology Company Ltd., Beijing, PR China) with 3% NaCl (pH 8.0), and then homogenized (BagMixer400, Interscience, France) for 2 min. Subsequently, the homogenate was serially diluted with APW and plated onto thiosulfate–citrate–bile salts–sucrose (TCBS,) to enumerate *V. parahaemolyticus* O3:K6 after incubation at 37 °C for 18–24 h. Three replicates at each sampling time were performed.

### 2.4. Latin Hypercube Sampling

LHS is a statistical technique that was developed by McKay [19] et al. that offers the benefits of efficient space filling and the capacity to match nonlinear connections. As a result, in this investigation, sample points were created using LHS.

### 2.5. Risk Reduction Assessment

**Figure 1:**
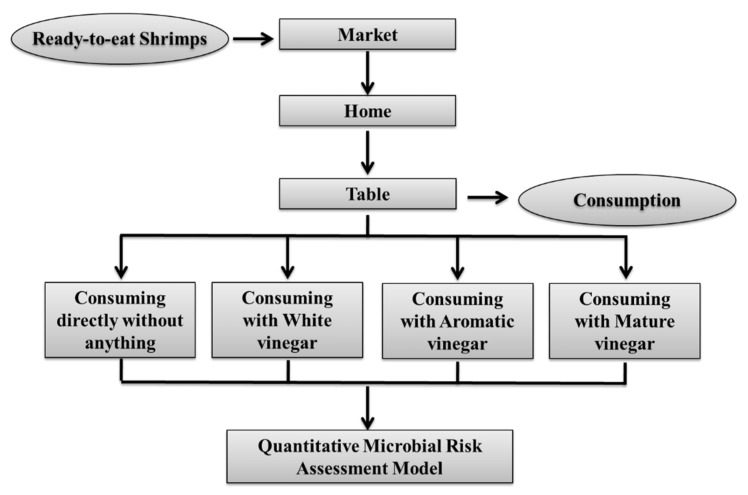
Diagram of the quantitative risk reduction assessment model for *V. parahaemolyticus* on ready-to-eat shrimp. The figure shows stages and their inputs from market to table. The input parameters of each stage are described in Table 2.

A simplified QMRA model was used in this study to evaluate the risk reduction of *V. parahaemolyticus* on ready-to-eat shrimp by consuming three different vinegars. The scope used in the QMRA model from market to table was shown in Figure 1, and the variables and models were listed in Table 2.

A uniform distribution was employed to describe the contamination of *V. parahaemolyticus* on ready-to-eat shrimp. The minimum of uniform distribution was 0, since we assumed there is no *Vibrio* on shrimp as the best circumstance. According to the previous researches, the most probable density of *V. parahaemolyticus* on shrimp was about 9 log CFU/g [20,21]. Hence, the 9 log CFU/g was chosen as the maximum of uniform distribution.

According to the surveillance research of Shanghai Food and Drug Administration (Shanghai FDA), the maximum of consumption of shrimp for one person per meal was approximately 43.74, and the minimum and maximum consumption was 10.85 g [22]. Hence, a normal distribution was used in this model for the possible shrimp consumption as normal (10.85, 43.74).

For appropriate assessment of the probability risk caused by a single cell of *V. parahaemolyticus*, a dose–response model was employed to estimate the risk of foodborne illness associated with shrimp consumption in the current research. Due to the lack of data in the city, the dose–response model used in this work was the same beta-Poisson model (Equation (1)) used by US FDA [17], including the distribution of uncertainty of parameters alpha and beta.

(1) $$P=1-{\left(1+\frac{D}{\beta }\right)}^{-\alpha }$$

where *P* denotes the probability of illness for an individual exposed to a certain dose (D cells); *D* is the number of *V. parahaemolyticus* consumed (CFU), *α* (0.60) and *β* (1.31 × 10^6^) are parameters of the dose–response.

The “Distribution Fitting” tool in @Risk software was employed to investigate the optimal distribution to describe the reduction of *V. parahaemolyticus* contamination level on ready-to-eat shrimp by three vinegars. In addition, the Chi-squared statistic, Anderson darling statistic, and Kolmogorov–Smirnov statistic were used to evaluate the goodness-of-fit of the distribution models.

### 2.6. Statistical Analyses

The statistical analyses were performed by using SPSS statistical package 17.0 (SPSS Inc., Chicago, IL, USA) with a significant level of 5% of probability. The model was developed by using a Microsoft^®^ Excel 2010 spreadsheet (Microsoft Corporation, Redmond, WA, USA). All simulations were run by using the Monte Carlo sampling method of input variables and combining the values properly in order to generate the output variables. The simulations were implemented by using the @Risk software. The distribution of uncertainty for the probability of disease was determined by running 10,000 iterations.

## 3. Results

### 3.1. Reduction of V. parahaemolyticus on Shrimp by Three Vinegars

The bactericidal effects of three vinegars (white vinegar, aromatic vinegar, and mature vinegar) on *V. parahaemolyticus* on ready-to-eat shrimp were experimented in the laboratory. According to the Chinese eating habit, the treatment times of 5 s to 120 s was chosen to investigate in this study, and the results of the effect of white vinegar, aromatic vinegar, and mature vinegar on *V. parahaemolyticus* on ready-to-eat shrimp can be found in Table 3.

**Figure 2:**
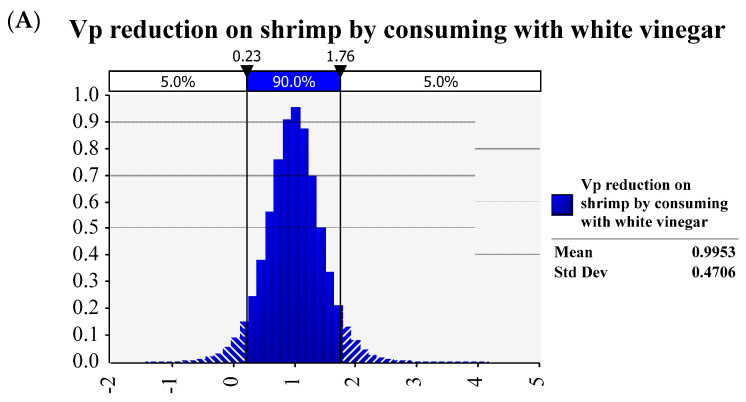

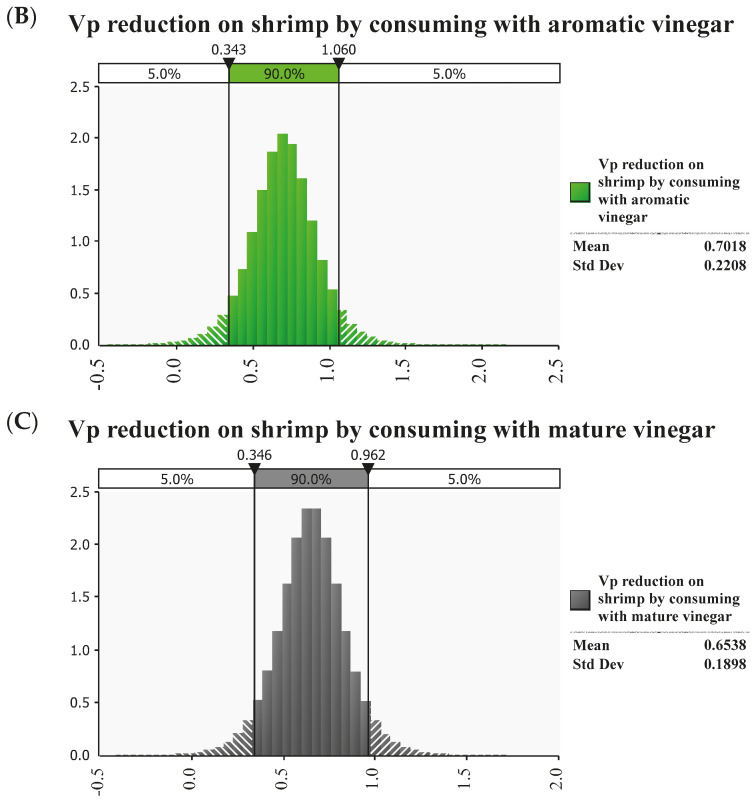
The reduction of *V. parahaemolyticus* contamination level on ready−to−eat shrimp by consuming with white vinegar (**A**), aromatic vinegar (**B**) and mature vinegar (**C**). Note: Vp = *Vibrio parahaemolyticus.*

The distributions of decreased contamination of *V. parahaemolyticus* for vinegars were fitted by @Risk software. The adequacy of the adjustment of data to a distribution of probability was evaluated by Chi-squared statistic, Anderson darling statistic, and Kolmogorov–Smirnov statistic using @Risk software. Figure 2 shows the optimal distribution for description of the reduction of *V. parahaemolyticus* contamination level on shrimp by three vinegars. The mean reduction of *V. parahaemolyticus* on ready-to-eat shrimp by consuming with white vinegar was 0.9953 log CFU/g (90% confidence interval 0.23 to 1.76). The mean reduction of *V. parahaemolyticus* on ready-to-eat shrimp by consuming with aromatic vinegar was 0.7018 log CFU/g (90% confidence interval 0.3430 to 1.060). The mean reduction of *V. parahaemolyticus* on ready-to-eat shrimp by consuming with mature vinegar was 0.6538 log CFU/g (90% confidence interval 0.3460 to 0.9620).

### 3.2. Final Contamination Level of V. parahaemolyticus on Shrimp after Consuming with Vinegars

**Figure 3:**
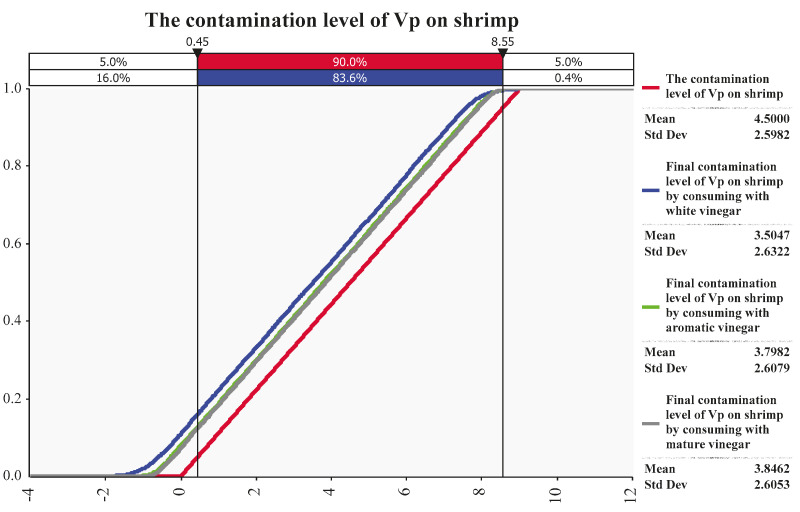
The final contamination level of *V. parahaemolyticus* on ready−to−eat shrimp by consuming without anything (Red Line), and with white vinegar (Blue Line), aromatic vinegar (Green Line) and mature vinegar (Grey Line). Note: Vp = *Vibrio parahaemolyticus*.

As shown in Figure 3, a uniform distribution was employed to describe the contamination of *V. parahaemolyticus* on ready-to-eat shrimp. The uniform distribution showed the mean contamination of *V. parahaemolyticus* on ready-to-eat shrimp assumed in this study was 4.50 log CFU/g with the standard deviation of 2.5982.

According to fitting results of @Risk software, after consuming with white vinegar, the mean contamination of *V. parahaemolyticus* on shrimp decreased as 3.5047 log CFU/g with the standard deviation of 2.6322. After consuming with aromatic vinegar, the mean contamination of *V. parahaemolyticus* on shrimp decreased as 3.7982 log CFU/g with the standard deviation of 2.6079. After consuming with mature vinegar, the mean contamination of *V. parahaemolyticus* on shrimp decreased as 3.8462 log CFU/g with the standard deviation of 2.6053.

### 3.3. Estimated Infection Risk Reductions from the QMRA

**Figure 4:**
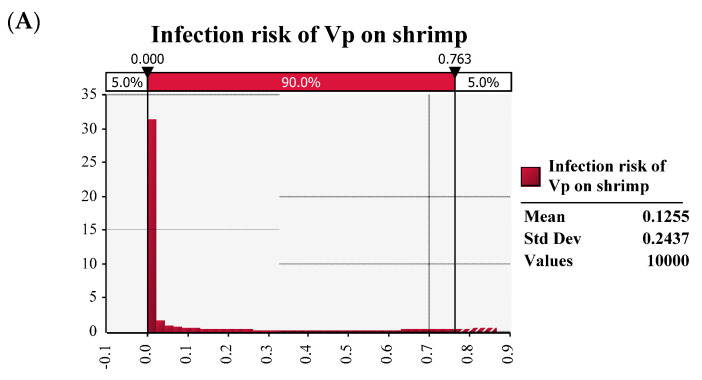

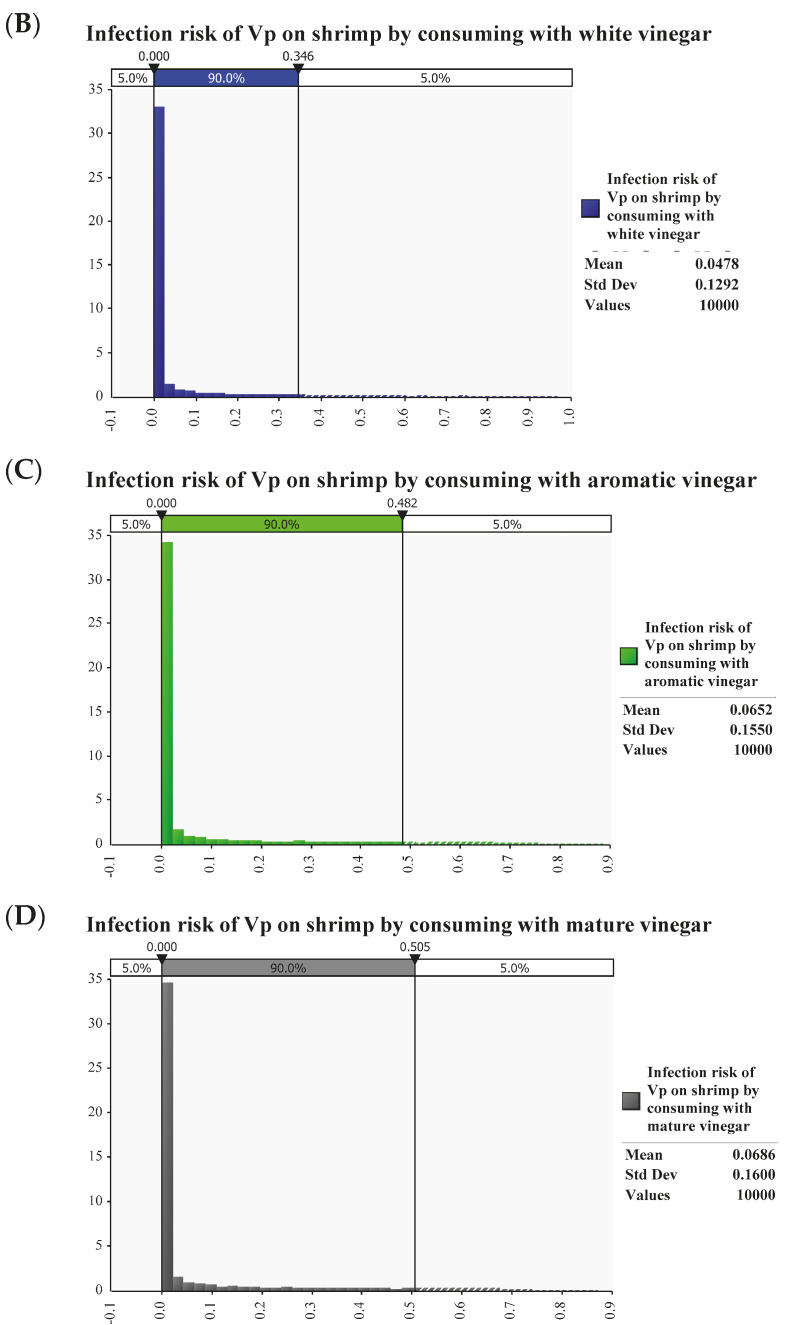
The infection risk of *Vibrio parahaemolyticus* on ready−to−eat shrimp by consuming without anything (**A**), and with white vinegar (**B**), aromatic vinegar (**C**) and mature vinegar (**D**).

The ingested dose of a single pathogenic organism was translated into the probability of illness using the dose–response model. Latin Hypercube sampling method was run for each simulated scenario to estimate the *V. parahaemolyticus* most probable final load and the probable number of illnesses after the complete process. Histograms depicting distributions of risk was shown in Figure 4, and the distributions approached their upper limit of one, and therefore had long right tails.

The infection risk of *V. parahaemolyticus* per meal in this QMRA model was quantified by a mean of 0.1250 with the standard deviation of 0.2437. However, after consuming with white vinegar, the mean infection risk of *V. parahaemolyticus* on shrimp decreased as 0.0478 with the standard deviation of 0.1292. After consuming with aromatic vinegar, the mean infection risk of *V. parahaemolyticus* on shrimp decreased as 0.0652 with the standard deviation of 0.1550. After consuming with mature vinegar, the mean infection risk of *V. parahaemolyticus* on shrimp decreased as 0.0686 with the standard deviation of 0.1600. Particularly, consuming with white vinegar is the most effective scenario to reduce the potential risk, where the proportion of risk reduction is high up to 61.76%.

## 4. Discussion

Today, the ready-to-eat seafood, such as ready-to-eat shrimp, fish steak, crab sticks, etc., become more and more popular by consumers since they are convenient and delicious [23]. However, this type of food is thought to be extremely prone to microbial pathogen contamination and might be dangerous to consumer health [24,25]. Human health is greatly concerned about the incidence and presence of foodborne pathogens in food, which can lead to illness outbreaks [26,27]. In shrimp that has been prepared for consumption, a number of foodborne viruses, most notably *V. parahaemolyticus* [28], may persist if no heat treatment step or other curing action is taken. A traditional viewpoint in China advocated pairing or mixing fish with vinegar to lower the danger of eating infectious microorganisms. However, up to now, this empirical traditional Chinese eating habit has not yet been proved scientifically. Therefore, this is the first study to evaluate the availability of a Chinese eating habit to reduce the infection risk of human from ready-to-eat shrimp contaminated with *V. parahaemolyticus* based on QMRA.

In China, the history of vinegar is well documented [29,30]. Vinegar serves as a medicine as well as a condiment [31]. White vinegar, aromatic vinegar, and mature vinegar were the three Chinese traditional vinegars used for this investigation. The treatment times of 5, 10, 15, 30, 60, 90, and 120 s were chosen to correspond to the Chinese eating custom. The “Distribution Fitting” feature in the @Risk program was used to match the best distribution for characterizing the decrease in *V. parahaemolyticus* contamination level on shrimp caused by the use of three vinegars. The findings indicated that the logistic distribution may well reflect the trend of three vinegars in reducing the amount of *V. parahaemolyticus* on shrimp. White vinegar, aromatic vinegar, and mature vinegar consumption resulted in a 0.9953 log CFU/g, 0.7018, and 0.7018 log CFU/g mean decrease in *V. parahaemolyticus* on ready-to-eat shrimp, respectively. This showed that this Chinese eating style can lower the amount of *V. parahaemolyticus* on shrimp that are ready to eat, and pairing it with white vinegar would have the biggest impact.

Within this QMRA model, the infection risk of *V. parahaemolyticus* per meal was quantified by a mean of 0.1250 and a standard deviation of 0.2437. That is significantly greater than what has been reported in previous studies, such as those on bloody clams in Malaysia [32] and Thailand [14], and horse mackerel in Japan [15], where the estimated risks were, respectively, 4.8 × 10^−6^, 5.6 × 10^−4^, and 5.6 × 10^−6^ to 1.4 × 10^−4^. This result was obtained because the worst case scenario (9 log CFU/g) was taken into account in this investigation. When researchers cannot precisely estimate the risk, they frequently utilize the worst-case scenario in a QMRA model [33,34]. In this method, researchers can constantly focus on the worst case scenario rather than having to think about the effects of uncertainty. As we can see, even though the worst-case scenario (9 log CFU/g) was chosen, the QMRA scenarios still showed that there were substantial decreases in the risk of infection when ingesting shrimp that had been prepared with vinegar. More precisely, the mean infection risk of *V. parahaemolyticus* on shrimp fell as 0.0478, 0.0652, and 0.0686 following consumption with white vinegar, aromatic vinegar, and mature vinegar, respectively.

Based on the above analysis, our study simulated the effects of vinegar on pathogenic microorganisms in seafood through a simplified quantitative microbial risk assessment model. Despite the lack of validation of epidemiological data of *V. parahaemolyticus*, comprehensive insights can be obtained that consuming with vinegar can reduce the infection risks from shrimp derived *V. parahaemolyticus*. Similar results were found in some published literatures, e.g., consuming sashimi with mustard had an effectively antimicrobial effect [35]. This Japanese eating habit can also reduce the infection risk of foodborne disease. That indicated a good eating habit will contribute the human health. In a summary, consuming seafood mixed or matched with vinegar was proved as a good eating habit to reduce the risk of bacterial infection, which should be suggested as a healthy dietary culture to spread around the world.

## Figures and Tables

**Table 1:** The major components of three vinegars.

Condiment	Total Acid Content (g/100 mL)	pH	Active Compounds
White vinegar	>6.00	2.59 ± 0.01	Polyphenols, organic acid
Aromatic vinegar	~5.75	3.82 ± 0.17	Polyphenols, organic acid
Mature vinegar	>6.00	3.48 ± 0.19	Polyphenols, organic acid

**Table 2:** Description and distribution of variables and models for risk reduction assessment of *V. parahaemolyticus* on ready-to-eat shrimp.

Variables	Definition	Unit	Assumption/Formula/Distribution	Source
C0	The contamination level of Vp on shrimp	log CFU/g	Uniform(0, 9)	Assumption
R1	Vp reduction on shrimp by consuming with white vinegar	log CFU/g	Logistic(0.99525, 0.25934)	Fitted by @Risk
R2	Vp reduction on shrimp by consuming with aromatic vinegar	log CFU/g	Logistic(0.70173, 0.12168)	Fitted by @Risk
R3	Vp reduction on shrimp by consuming with mature vinegar	log CFU/g	Logistic(0.65378, 0.10464)	Fitted by @Risk
C1	Final contamination level of Vp on shrimp by consuming with white vinegar	log CFU/g	C0–R1	Calculated
C2	Final contamination level of Vp on shrimp by consuming with aromatic vinegar	log CFU/g	C0–R2	Calculated
C3	Final contamination level of Vp on shrimp by consuming with mature vinegar	log CFU/g	C0–R3	Calculated
S	Consumption of shrimp per meal	g	Normal (10.85, 43.74)	Shanghai FDA
P	Infection risk of Vp on shrimp		1 − [1 + (10^C0^ × S)/(27 × 1.31 × 10^6^)]^−0.6^	Calculated
P1	Infection risk of Vp on shrimp by consuming with white vinegar		1 − [1 + (10^C1^ × S)/(27 × 1.31 × 10^6^)]^−0.6^	Calculated
P2	Infection risk of Vp on shrimp by consuming with aromatic vinegar		1 − [1 + (10^C2^ × S)/(27 × 1.31 × 10^6^)]^−0.6^	Calculated
P3	Infection risk of Vp on shrimp by consuming with mature vinegar		1 − [1 + (10^C3^ × S)/(27 × 1.31 × 10^6^)]^−0.6^	Calculated

Note: a Vp = *Vibrio parahaemolyticus*; The distributions in Table 2 were performed according to Guide to Using @RISK-Risk Analysis and Simulation Add-In for Microsoft^®^ Excel Version 5.7, Palisade Corporation.

**Table 3:** Inhibitory effect of three vinegars to shrimp-derived *V. parahaemolyticus.*

Treatment Time	Chinese Traditional Vinegar (log CFU/g)		
	White Vinegar	Aromatic Vinegar	Mature Vinegar
5 s	0.27 ± 0.05 ^a^	0.20 ± 0.22 ^ab^	0.25 ± 0.15 ^a^
10 s	0.74 ± 0.27 ^b^	0.56 ± 0.40 ^ab^	0.55 ± 0.23 ^ab^
15 s	0.94 ± 0.23 ^b^	0.69 ± 0.02 ^a^	0.58 ± 0.18 ^b^
30 s	0.97 ± 0.10 ^b^	0.71 ± 0.16 ^ab^	0.65 ± 0.11 ^ab^
60 s	1.04 ± 0.09 ^b^	0.77 ± 0.04 ^ab^	0.75 ± 0.33 ^b^
90 s	1.25 ± 0.44 ^b^	0.81 ± 0.21 ^ab^	0.81 ± 0.10 ^ab^
120 s	1.97 ± 0.03 ^b^	0.98 ± 0.29 ^b^	0.84 ± 0.10 ^b^

Mean values (*p* < 0.05). Values followed by different lowercase letters in the same row indicate significant differences during treatment.

## Data Availability

All datasets generated for this study are included in the article.
